# ADAM33 polymorphisms are associated with asthma and a distinctive palm dermatoglyphic pattern

**DOI:** 10.3892/mmr.2013.1733

**Published:** 2013-10-14

**Authors:** WEILIN XUE, WEI HAN, ZHAO-SHAN ZHOU

**Affiliations:** 1 Department of Respiratory Medicine, Qingdao Haici Hospital, Qingdao, Shandong 266033, P.R. China; 2 Department of Respiratory Medicine, Qingdao Municipal Hospital, Qingdao, Shandong 266071, P.R. China

**Keywords:** asthma, a disintegrin and metalloprotein-33, palm, polymorphisms, dermatoglyphics

## Abstract

A close correlation between asthma and palm dermatoglyphic patterns has been observed in previous studies, but the underlying genetic mechanisms have not been investigated. A disintegrin and metalloprotein-33 (*ADAM33*) polymorphisms are important in the development of asthma and other atopic diseases. To investigate the underlying mechanisms of the association between asthma and distinctive palm dermatoglyphic patterns, thirteen ADAM33 single-nucleotide polymorphisms (SNPs) were analyzed for the association between asthma and palm dermatoglyphic patterns in a population of 400 asthmatic patients and 200 healthy controls. Based on the results, five SNPs, rs44707 (codominant model, P=0.031; log-additive model, P=0.0084), rs2787094 (overdominant model, P=0.049), rs678881 (codominant model, P=0.028; overdominant model, P=0.0083), rs677044 (codominant model, P=0.013; log-additive model, P=0.0033) and rs512625 (dominant model, P=0.033), were associated with asthma in this population. Two SNPs, rs44707 (dominant model, P=0.042) and rs2787094 (codominant model, P=0.014; recessive model, P=0.0038), were observed in the asthma patients with the distinctive palm pattern. As rs44707 and rs2787094 are associated with asthma and a distinctive palm pattern, the data suggest that ADAM33 polymorphisms are correlated with asthma and may be the underlying genetic basis of the association between asthma and palm dermatoglyphic patterns.

## Introduction

Asthma is a multi-factorial disorder resulting from a combination of genetic and environmental factors. As genetic changes are not only involved in the pathogenesis of asthma, but also the response to asthma treatment, it may be valuable to identify key genes in asthmatic individuals (1,2). However, it is impossible to detect all genes for every asthma patient due to the expensive and time-consuming detection process. Thus, certain simple and practical makers are used to recognize the different phenotypes for the clinical management of asthma.

Dermatoglyphs, patterns of skin ridges, are derived from the hypodermal neural system and formed embryologically between the 10th and 17th weeks. Similar to other physical features, dermatoglyphs are affected by genes and the environment, and are particularly prevalent in primates (3). Due to the original observation that individuals affected with Down's syndrome possess abnormal palm prints, a series of studies have been performed concerning the possible association between an altered dermatoglyphic pattern and certain congenital defects or gene-related diseases (4–6). However, due to the complex influence of different factors on dermatoglyphic traits, such as age-related changes, no practical association has been confirmed in human beings; and even sex and ethnic differences exhibited a substantial effect on fingerprint and palmar ridge density in certain studies (7,8). Recently, several genetic modes of inheritance for certain dermatoglyph indices and genomic linkages between palmer ridge counts and chromosomes were demonstrated (9,10). A possible association of finger print patterns with children suffering from acute lymphoblastic leukemia have also been investigated (11). These findings have prompted studies to investigate gene-based dermatoglyphic markers, which may aid in the diagnosis and prognosis of diseases.

In our previous study, a particular palm pattern was observed to be prevalent in asthmatic patients, which is characterized by an increased ridge count (>10) and a deep grid pattern in the thenar area (12). Recently Mahajan *et al*(13) and Pakhale *et al*(14) analyzed the qualitative dermatoglyphic parameters, such as whorls, loops and arches, on the hands of asthmatic patients, and identified some differences on the palms of asthma patients compared with those of the controls (13,14). However, the genomic basis of the association between dermatoglyphs and asthma has rarely been investigated.

In the past 20 years, an increasing number of gene loci predisposing asthma and other atopic disorders have been identified (15). Among these genes, a disintegrin and metalloprotein-33 (*ADAM33*), the first asthma candidate gene identified by positional cloning on human chromosome 20p, is important in asthma and bronchial hyperresponsiveness (16). With the completion of the Human Genome Project, analysis of single-nucleotide polymorphisms (SNPs) has become the newest approach in the detection and localization of genetic determinants of human disease. In the last 20 years, >300 SNPs of ADAM33 have been identified in humans and the majority of these are associated with asthma and allergic diseases (17,18). However, due to the differences in genes between ethnic groups, ADAM33 polymorphisms in East Chinese populations and its correlation with palm dermatoglyphic patterns in asthmatic patients remains to be investigated. In the present study thirteen SNPs of ADAM33 were analyzed in an Eastern Chinese population, particularly in asthma patients with the distinct palm pattern, to clarify the ADAM33 association with asthma and the genetic basis of the association between asthma and palm dermatoglyphs.

## Materials and methods

### Subjects

Two hundred control subjects and 400 asthmatics of Han nationality were recruited from adult respiratory clinics of two teaching hospitals, Qingdao Haici Hospital and Qingdao Municipal Hospital of China (Qingdao, China). Asthma was diagnosed based on symptoms and spirometry assessments using the criteria outlined by the American Thoracic Society according to the Global Initiative for Asthma (1). Control subjects were asymptomatic for asthma and were devoid of atopic or pulmonary diseases. Pregnant or lactating female subjects were excluded. The study was in accordance with the Helsinki Declaration and was approved by the local ethics committee of Qingdao Haici Hospital and the Municipal Hospital (Qingdao, China). All subjects provided written informed consent prior to the study.

### Dermatoglyphic palm pattern prints

**Figure 1:**
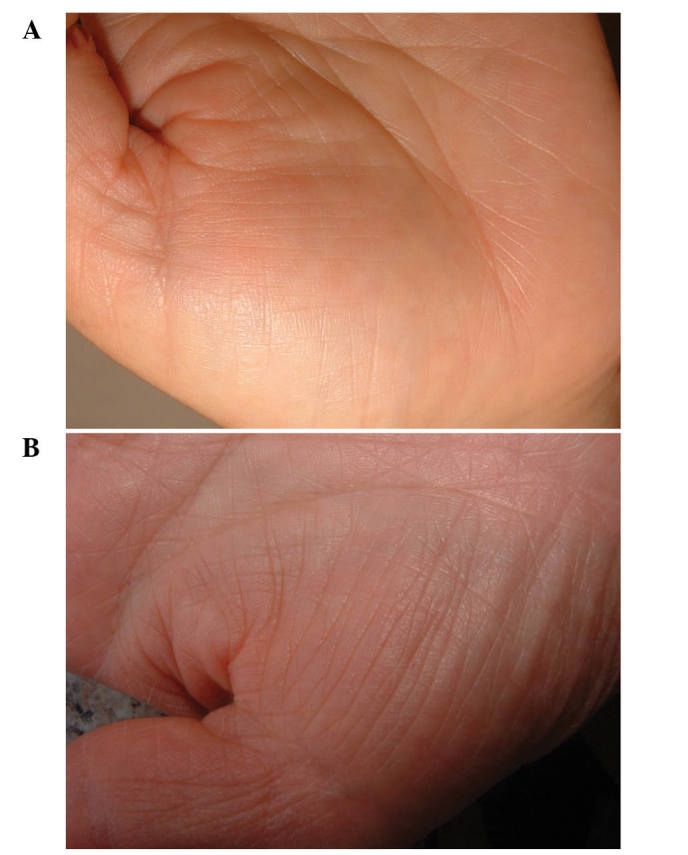
(A) Hand of a 71-year-old healthy female control whose palm dermatoglyphic was smooth with a few superficial grips in the greater thenar. (B) Hand of a 68-year-old female asthma patient whose palm dermatoglyph was rough and with numerous deep grips in the greater thenar.

The palms were observed with the naked-eye following washing and cleaning with soap and water. The dermatoglyphic variables, ridge count (number of ridges) and shape recognition (shape of the ridge), in the thenar area were counted and recorded. Compared with the palm dermatoglyphic pattern of the healthy control, the palm dermatoglyphic pattern of asthmatics was distinctive and typically exhibited an increased ridge count (>10) and a deep grid pattern in the thenar area (Fig. 1). All subjects were sub-categorized into two groups, the positive and negative palm pattern groups, according to the presence of the distinctive palm pattern.

### Polymorphism genotyping

Genomic DNA was isolated from peripheral blood leukocytes using a DNA extraction kit (Tiangen, Beijing, China). Based on the results of previous studies, thirteen ADAM33 polymorphisms genotyped in the 3′ region from exon 19 to 22 were selected. The PCR primers were designed as shown in Table I.

PCR amplification of the corresponding genomic region surrounding each SNP locus was performed in a Takara PCR thermal cycler (Takara TP600, Dalian, China). The reaction was performed in a final volume of 10 μl, which contained 3.0 mM Mg^2+^, 0.3 mM dNTP, 1X HotStarTaq polymerase (Qiagen Inc., Hilden, Germany), 1 μl each primer and 1 μl (10 ng) genomic DNA. Cycling conditions were as follows: One cycle at 95°C for 2 min; 11 cycles at 94°C for 20 sec; 65-0.5°C for 40 sec, 72°C for 1.5 min; and 24 cycles at 94°C for 20 sec, 59°C for 30 sec and 72°C for 1.5 min; and a final extension at 72°C for 2 min. PCR products were purified by the PCR purification kit containing 1X shrimp alkaline phsophatase (SAP) and 1X Exonuclease I (Qiagen, Hilden, Germany) and were used as DNA templates for cycle sequencing. Direct DNA sequencing was performed using 5 μl SNaPshot Multiplex kit (ABI) in 10-μl volumes containing 2 μl primer and 2 μl DNA template. Samples were subjected to one cycle at 96°C for 1 min, 28 cycles of denaturation at 96°C for 10 sec, annealing at 52°C for 5 sec and extension at 60°C for 30 sec. Sequencing products were purified by the 1X SAP at 37°C for 1 h, and annealing occured at 75°C for 15 min. All SNPs were detected by ABI3130XL (Applied Biosystems Co., Ltd., Carlsbad, CA, USA) and the data were analyzed with GeneMapper 4.0 (Applied Biosystems Co., Ltd.). The association analysis between single SNPs and phenotype was conducted using five different genetic models (inheritance patterns): codominant, dominant, recessive, overdominant and log-additive models.

### Statistical analysis

The Hardy-Weinberg equilibrium was estimated using the χ^2^ test. Differences in genotype distribution between the asthmatic patients and healthy controls were analyzed using the χ^2^ test. P≤0.05 was considered to indicate a statistically significant difference. All statistical analyses were performed using SPSS version 17.0 software (SPSS Inc., Chicago, IL, USA).

## Results

### Demography and palm pattern

The demographic features and palm patterns in asthma patients and controls are shown in Table II. The mean duration of asthma for asthmatic patients was 11.35 years. Cases and controls exhibited similar age and sex distributions (P=0.25 and P=0.31, respectively), but the incidence of the distinct palm pattern in asthmatic patients was higher than that in the controls (P<0.01).

### Association between ADAM33 polymorphism and asthma

All genotype frequencies were consistent with Hardy-Weinberg equilibrium (P>0.05). As shown in Table III, there were only five ADAM33 SNPs identified with statistically significant differences between the asthma group and the control group in genotype distribution (P<0.05). These are rs44707 (codominant model: P=0.031; log-additive model: P=0.0084), rs678881 (codominant model: P=0.028; overdominant model: P=0.0083), rs677044 (codominant model: P=0.013; log-additive model: P=0.0033), rs512625 (dominant model: P=0.033) and rs2787094 (overdominant model: P=0.049).

### Association between ADAM33 polymorphisms and palm patterns

As shown in Table IV, only two ADAM33 genotypes, rs44707 and rs2787094, were correlated with the positive palm pattern in different models (rs44707: dominant model; rs2787094: codominant or recessive model, P<0.05). Notably, the two ADAM33 genotypes, rs44707 and rs2787094, were also associated with asthma, which indicates the ADAM33 polymorphism may be the genetic basis of the association between asthma and the distinct palm pattern.

## Discussion

The gene segments were amplified covering thirteen ADAM33 SNPs in a population of 400 asthmatics and 200 healthy controls to investigate the genotype of asthmatic patients with a distinct palm pattern in the study population from Eastern China.

As a member of the ADAM family, ADAM33 has been implicated in a variety of biological processes involving cell-cell and cell-matrix interactions, including fertilization, muscle development and neurogenesis. Howard *et al*(19) first reported an association between ADAM33 and asthma in ethnically diverse populations and replication studies in subjects derived from different populations in Europe, America and Asia have also been conducted (17,20–24). However, certain studies in other populations were not able to confirm the association between ADAM33 polymorphisms and asthma (25,26). In the present study, five ADAM33 SNPs were associated with asthma in the study population using a different model. Among the five SNPs, three (rs2787094, rs44707 and rs512625) have been demonstrated to be associated with asthma in other populations; rs2787094 and rs44707 have been shown to be key in the development and severity of asthma in different populations since 2006 (26,27). They were considered to be candidate genes for other chronic inflammatory diseases, including psoriasis and type 1 diabetes (28). Bukvic *et al*(29) demonstrated a significant interaction between rs512625 and early-life environmental tobacco smoke exposure in association with hospitalization (P=0.02) and lung function (P=0.03) (29). In order to investigate other possible ADAM33 polymorphisms in the present study population, six novel ADAM33 SNPs were detected. Two SNPs, rs678881 and rs677044 were shown to be associated with asthma in this Eastern Chinese population. The results confirmed a significant involvement of ADAM33 polymorphisms in the development of asthma in the study population. However, notably the 13 SNPs in the current study only represent a proportion of the total variation of the gene, thus is is necessary to identify more ADAM33 SNPs in other populations and functionally analyze these polymorphisms particularly in regard to the molecular mechanisms of asthma.

Dermatoglyphics, the study of the patterns of ridges on the skin of the fingers, palms, toes and soles, is of interest in the field of anthropology, criminology and medicine (3). In the medical field, dermatoglyphics is involved in dysmorphology, such as trisomy 21, and certain genetic diseases, such as schizophrenia and Alzheimer's disease. In the previous study, it was demonstrated that a typical asthmatics palm, characterized by numerous deep grids in thenar area of the hands, was correlated with the duration and treatment response of asthma. Recently, Pakhale *et al*(14) further demonstrated that numerous dermatoglyphic parameters, such as the number of arches and finger ridges, changed in asthma patients compared with the controls. Therefore, palm dermatoglyphics were observed to be a biomarker for asthma; however, the underlying mechanisms for the association between dermatoglyphs and asthma remain poorly understood. As described previously, several studies have demonstrated a genetic basis of the association between dermatoglyphs and certain diseases (4–6,11). As asthma is also a type of genetic disease, it is suggested that there may be a common genetic basis for the distinct palm pattern in asthmatics. To investigate this hypothesis, the association between *ADAM33* SNPs and palm dermatoglyphic patterns was observed in this study. Among the thirteen *ADAM33* SNPs investigated, only two *ADAM33* SNPs, rs44707 and rs2787094, were significantly different between the different palm pattern groups. As these two *ADAM33* SNPs have been demonstrated to be correlated with asthma in the study population, they may demonstrate a genetic basis for the association between asthma and the palm dermatoglyphic pattern.

In conclusion, the results confirmed the involvement of ADAM33 polymorphisms in the asthmatic patients, particularly in the patients with a distinctive palm dermatoglyphic pattern. Therefore, this study aimed to investigate the genetic basis of the association between asthma and the typical palm pattern for the first time. It was suggested that in asthma patients the distinctive palm pattern was considered to be a biomarker for ADAM33 polymorphisms, which may aid in the development of a therapeutic strategy. Further population and functional studies are required to elucidate the exact mechanism and functional effects of ADAM33 variants on asthma.

## Figures and Tables

**Table I:** Oligonucleotide primers used for resequencing the 3′ region of ADAM33.

SNP	Oligonucleotide primers
rs2280090	F: GGGGAGGCAATAACCCACTCAG R: TGACTGGGTGCTGCCCATCT
rs487377	F: CTGGGCTGTGACCAAGAGGAGA R: CCTGGTACCCTGCCCCTTGATA
rs511898	F: ATGACCAGCCTTCCTGGTGGT R: ACTGGGACTCGAGGCCTGTGAA
rs2787094	F: GTCCTGGGGCCCTATGGTTC R: AATGGGGGAGAGGCTGTCAGAT
rs528557	F: GGTGCACCTGCTCAGGACTCA R: CTCCTGGGAGTCGGTAGCAACA
rs678881	F: TTCAAAGCCTCCCCCTCTCATC R: TGATCCAGGAAAAGCCACAGC
rs2853215	F: CTCATCTCCTCCCCTGCAACCT R: GCCACATGGGGATCAGAGTCAT
rs554743	F: TGGATTCTCGCTCTCACAACCAG R: GCCTGAGAGCAAAGCAGGGACT
rs2853209	F: CCCAGAGGCCATGGAAAGAAGT R: GGGAGAAGCAGGAGAGTGGACA
rs570269	F: GCCTGAACCTTCAGCCTCACTG R: CAGAAGAAGCACATGGGGTGGT
rs44707	F: TTTTCCCTGGCTCAGATTGCAG R: GCCCAGCATTTGGGAACTTCA
rs512625	F: GGTGGCTGACGGGGTGGT R: TGGTTTTGTTATGCGGCAACAG
rs677044	F: TGCAGGCAGCTTGGAAGTTTCT R: AGTGTGCCCAGCAGTGTTCTCC

ADAM33, a disintegrin and metalloprotein-33; SNP, single nucelotide polymorphism.

**Table II:** Palm pattern in asthmatic patients and healthy controls.

Distinct palm pattern	Controls	Asthma Patients	Total
Negative (%)	180 (90.0)	139 (34.8)	319 (53.2)
Positive (%)	20 (10.0)	261 (65.3)	281 (46.8)
Pearson's χ^2^		163.459	
P-value		<0.01	

**Table III:** Association of ADAM33 SNPs with asthma.

Model	Genotype	Controls (%)	Asthma Patients (%)	OR (95% CI)	P-value
rs2280090	C/C	166 (83)	331 (83)	1	0.33
	C/T	34 (17)	65 (16.3)	1.03 (0.61–1.71)	
	T/T	0 (0)	3 (0.8)	NA (0.00-NA)	
rs487377	G/G	82 (41)	142 (35.6)	1	0.79
	G/A	90 (45)	197 (49.4)	1.15 (0.76–1.75)	
	A/A	28 (14)	60 (15)	1.03 (0.57–1.88)	
rs511898	G/G	97 (48.5)	185 (46.2)	1	0.94
	G/A	87 (43.5)	179 (44.8)	1.04 (0.70–1.55)	
	A/A	16 (8)	36 (9)	1.13 (0.55–2.31)	
rs2787094	C/C	90 (45)	143 (35.8)	1	0.049
	C/G	89 (44.5)	189 (47.2)	1.21 (0.80–1.82)	
	G/G	21 (10.5)	68 (17)	1.91 (1.02–3.60)	
rs528557	G/G	124 (62)	248 (62)	1	0.56
	G/C	66 (33)	133 (33.2)	0.96 (0.63–1.44)	
	C/C	10 (5)	19 (4.8)	1.61 (0.63–4.14)	
rs678881	C/C	166 (83)	298 (74.7)	1	0.0083
	G/C	30 (15)	97 (24.3)	1.92 (1.16–3.18)	
	G/G	4 (2)	4 (1)	0.68 (0.14–3.38)	
rs2853215	C/C	128 (64)	250 (62.5)	1	0.39
	C/T	64 (32)	127 (31.8)	1.02 (0.67–1.54)	
	T/T	8 (4)	23 (5.8)	1.91 (0.73–5.01)	
rs554743	A/A	64 (32.3)	122 (30.5)	1	0.51
	G/A	100 (50.5)	193 (48.2)	0.84 (0.54–1.30)	
	G/G	34 (17.2)	85 (21.2)	1.10 (0.63–1.94)	
rs2853209	A/A	43 (21.5)	108 (27.2)	1	0.37
	A/T	102 (51)	201 (50.6)	0.82 (0.51–1.33)	
	T/T	55 (27.5)	88 (22.2)	0.67 (0.39–1.17)	
rs570269	G/G	66 (33)	140 (35)	1	0.49
	G/C	93 (46.5)	199 (49.8)	1.01 (0.66–1.55)	
	C/C	41 (20.5)	61 (15.2)	0.74 (0.43–1.29)	
rs44707	C/C	66 (33)	172 (43)	1	0.0084
	C/A	90 (45)	178 (44.5)	0.73 (0.47–1.12)	
	A/A	44 (22)	50 (12.5)	0.47 (0.27–0.83)	
rs512625	C/C	94 (47)	142 (35.5)	1	0.033
	C/T	75 (37.5)	193(48.2)	1.60 (1.06–2.43)	
	T/T	31 (15.5)	65 (16.2)	1.32 (0.74–2.34)	
rs677044	T/T	120 (60)	193 (48.6)	1	0.0033
	C/T	69 (34.5)	167 (42.1)	1.51 (1.00–2.28)	
	C/C	11 (5.5)	37 (9.3)	2.77 (1.23–6.24)	

ADAM33, a disintegrin and metalloprotein-33; SNPs, single-nucleotide polymorphisms.

**Table IV:** Association of ADAM33 SNPs with the distinctive asthmatic palm pattern.

Model	Genotype	Negative palm (%)	Positive palm (%)	OR (95% CI)	P-value
rs2280090	C/C	261 (81.8)	236 (84.3)	1	0.11
	C/T	58 (18.2)	41 (14.6)	0.78 (0.49–1.24)	
	T/T	0 (0)	3 (1.1)	NA (0.00-NA)	
rs487377	G/G	120 (37.6)	104 (37.1)	1	0.95
	G/A	153 (48)	134 (47.9)	0.95 (0.65–1.38)	
	A/A	46 (14.4)	42 (15)	1.01 (0.59–1.73)	
rs511898	G/G	146 (45.8)	136 (48.4)	1	0.44
	G/A	147 (46.1)	119 (42.4)	0.80 (0.55–1.15)	
	A/A	26 (8.2)	26 (9.2)	1.01 (0.53–1.92)	
rs2787094	C/C	128 (40.1)	105 (37.4)	1	0.014
	C/G	155 (48.6)	123 (43.8)	0.92 (0.63–1.34)	
	G/G	36 (11.3)	53 (18.9)	2.00 (1.15–3.48)	
rs528557	G/G	197 (61.8)	175 (62.3)	1	0.43
	G/C	107 (33.5)	92 (32.7)	0.92 (0.63–1.33)	
	C/C	15 (4.7)	14 (5)	1.66 (0.69–3.99)	
rs678881	C/C	246 (77.1)	218 (77.9)	1	0.93
	G/C	68 (21.3)	59 (21.1)	0.93 (0.61–1.43)	
	G/G	5 (1.6)	3 (1.1)	1.16 (0.23–5.95)	
rs2853215	C/C	204 (64)	174 (61.9)	1	0.41
	C/T	101 (31.7)	90 (32)	1.08 (0.74–1.57)	
	T/T	14 (4.4)	17 (6)	1.73 (0.76–3.95)	
rs554743	A/A	93 (29.3)	93 (33.1)	1	0.16
	G/A	163 (51.4)	130 (46.3)	0.68 (0.46–1.02)	
	G/G	61 (19.2)	58 (20.6)	0.87 (0.53–1.44)	
rs2853209	A/A	72 (22.6)	79 (28.3)	1	0.16
	A/T	170 (53.5)	133 (47.7)	0.66 (0.43–1.02)	
	T/T	76 (23.9)	67 (24)	0.79 (0.48–1.31)	
rs570269	G/G	103 (32.3)	103 (36.6)	1	0.4
	G/C	156 (48.9)	136 (48.4)	0.88 (0.60–1.30)	
	C/C	60 (18.8)	42 (14.9)	0.70 (0.42–1.18)	
rs44707	C/C	115 (36)	123 (43.8)	1	0.042
	C/A	150 (47)	118 (42)	0.67 (0.46–0.98)	
	A/A	54 (16.9)	40 (14.2)	0.76 (0.45–1.30)	
rs512625	C/C	136 (42.6)	100 (35.6)	1	0.19
	C/T	136 (42.6)	132 (47)	1.27 (0.87–1.85)	
	T/T	47 (14.7)	49 (17.4)	1.59 (0.94–2.71)	
rs677044	T/T	168 (52.8)	145 (52)	1	0.45
	C/T	127 (39.9)	109 (39.1)	0.93 (0.65–1.34)	
	C/C	23 (7.2)	25 (9)	1.46 (0.73–2.91)	

ADAM33, a disintegrin and metalloprotein-33; SNPs, single-nucleotide polymorphisms. Negative and positive palm, indictaed the absence or presence of the distinct asthmaric palm pattern which typically exhbitied an increased ridge count (>10) and a deep grid pattern in the thenar area.
